# Crack propagation in cortical bone is affected by the characteristics of the cement line: a parameter study using an XFEM interface damage model

**DOI:** 10.1007/s10237-019-01142-4

**Published:** 2019-04-08

**Authors:** Anna Gustafsson, Mathias Wallin, Hanifeh Khayyeri, Hanna Isaksson

**Affiliations:** 1 Department of Biomedical Engineering, Lund University, Box 118, 221 00 Lund, Sweden; 2 Division of Solid Mechanics, Lund University, Box 118, 221 00 Lund, Sweden

**Keywords:** Osteons, Fracture toughness, Crack deflection, Interface, Microstructure

## Abstract

Bulk properties of cortical bone have been well characterized experimentally, and potent toughening mechanisms, e.g., crack deflections, have been identified at the microscale. However, it is currently difficult to experimentally measure local damage properties and isolate their effect on the tissue fracture resistance. Instead, computer models can be used to analyze the impact of local characteristics and structures, but material parameters required in computer models are not well established. The aim of this study was therefore to identify the material parameters that are important for crack propagation in cortical bone and to elucidate what parameters need to be better defined experimentally. A comprehensive material parameter study was performed using an XFEM interface damage model in 2D to simulate crack propagation around an osteon at the microscale. The importance of 14 factors (material parameters) on four different outcome criteria (maximum force, fracture energy, crack length and crack trajectory) was evaluated using ANOVA for three different osteon orientations. The results identified factors related to the cement line to influence the crack propagation, where the interface strength was important for the ability to deflect cracks. Crack deflection was also favored by low interface stiffness. However, the cement line properties are not well determined experimentally and need to be better characterized. The matrix and osteon stiffness had no or low impact on the crack pattern. Furthermore, the results illustrated how reduced matrix toughness promoted crack penetration of the cement line. This effect is highly relevant for the understanding of the influence of aging on crack propagation and fracture resistance in cortical bone.

**Electronic supplementary material:**

The online version of this article (10.1007/s10237-019-01142-4) contains supplementary material, which is available to authorized users.

## Introduction

Bone tissue is a complex material that adapts during the course of life, and structural and material changes occur at all hierarchical length scales of bone (Launey et al. 2010; Zimmermann et al. 2015). Old bone is characterized by reduced fracture toughness with compromised ability to slow down or deflect propagating cracks (Chan et al. 2009; Koester et al. 2011; Nalla et al. 2004, 2006; Zimmermann et al. 2011), increased porosity (Cooper et al. 2007; Mirzaali et al. 2016; Stein et al. 1999) and reduced plasticity at the nanoscale (Zimmermann et al. 2011). One detrimental effect of aging is the increased risk of fractures in patients with osteoporosis (Cummings and Melton 2002; Hernlund et al. 2013). Yet, it is very challenging to experimentally measure damage parameters locally (Kruzic et al. 2009) and to distinguish how the fracture resistance is affected by local material or structural alterations. Instead, existing methods measure average bulk behavior of cortical bone tissue (Koester et al. 2008, 2011; Nalla et al. 2005). Furthermore, crack deflections along cement lines are identified as important toughening mechanisms in cortical bone (Chan et al. 2009; Koester et al. 2008, 2011; Nalla et al. 2006; Zimmermann et al. 2009, 2010), but still there is no consensus regarding the mechanical properties of the interface (Burr et al. 1988; Milovanovic et al. 2018; Montalbano and Feng 2011; Skedros et al. 2005). At this point, computer models can improve the understanding of how microstructure contributes to tissue fracture toughness.

Traditionally, there have been two types of theoretical models for composite materials for analyzing crack propagation at an interface: strength-based models, as introduced by Cook and Gordon (1964), where the difference in strength between interface and substrate (i.e., osteon in the case of cortical bone) dictates whether the crack will be deflected by the interface, and energy-based models where the crack path is determined by the maximum energy release when comparing different possible crack paths (He and Hutchinson 1989). These ideas are combined in cohesive damage models that account for both strength and energy when modeling crack propagation. Parmigiani and Thouless (2006) used cohesive elements to show that both strength and energy can be important for the crack trajectory at an interface. Mischinski and Ural (2011) modeled crack propagation in cortical bone by outlining two possible crack paths with cohesive elements: one penetrating the osteon and the other deflecting along the cement line. They concluded that low cement line strength was the most critical factor for promoting crack deflection (Mischinski and Ural 2011).

Another option for modeling crack propagation is the extended finite element method (XFEM) which has the benefit, that is, it does not require a predefined crack path (Belytschko and Black 1999; Melenk and Babuska 1996). A handful of XFEM models have been used to model crack propagation in 2D at the microscale in cortical bone, and the maximum principal strain (MAXPE) criterion has been commonly used to model damage initiation with cohesive cracks (Abdel-Wahab et al. 2012; Budyn and Hoc 2007; Idkaidek and Jasiuk 2017; Li et al. 2013; Vergani et al. 2014). However, the MAXPE criterion cannot be used to model realistic crack trajectories around osteons, as it always predicts crack penetration of the cement line interface (Gustafsson et al. 2018a). To handle this problem, we recently proposed an XFEM interface damage model that is able to capture both crack deflections along cement line interfaces and crack propagation through osteons (Gustafsson et al. 2018a). Additionally, Marco et al. (2018a) proposed heterogeneous maximum tangential stress criteria to model crack propagation along cement lines. However, the lack of experimental data is reflected in the computational models, where material parameters governing crack propagation are loosely defined and based on assumptions. As an effect, *one* set of damage parameters (e.g., parameters used in Abdel-Wahab et al. 2012) has been used in almost all XFEM models simulating crack propagation in cortical bone. Furthermore, there is a large uncertainty regarding the cement line stiffness where values used in recent modeling studies range from more than 160 times lower (Marco et al. 2018a) to 20% higher (Gustafsson et al. 2018a) than the matrix stiffness. Hence, there is a need to thoroughly evaluate the parameters used to model crack propagation in cortical bone.

The aim of this study was therefore to identify the most important material parameters for crack propagation in cortical bone by evaluating their effect on the maximum force, fracture energy, crack length and crack trajectory. By identifying important material parameters numerically, this study will reveal what parameters need to be studied or measured with higher accuracy experimentally. As experimental studies show that the crack pattern at the microscale affects the fracture resistance of cortical bone tissue, e.g., (Chan et al. 2009; Koester et al. 2008), this study focused on the interactions between a propagating crack and an osteon. Crack propagation was simulated in 2D using simplified microstructural geometries depicting an osteon surrounded by a cement line interface in different orientations (Gustafsson et al. 2018a).

## Methods

**Fig. 1:**
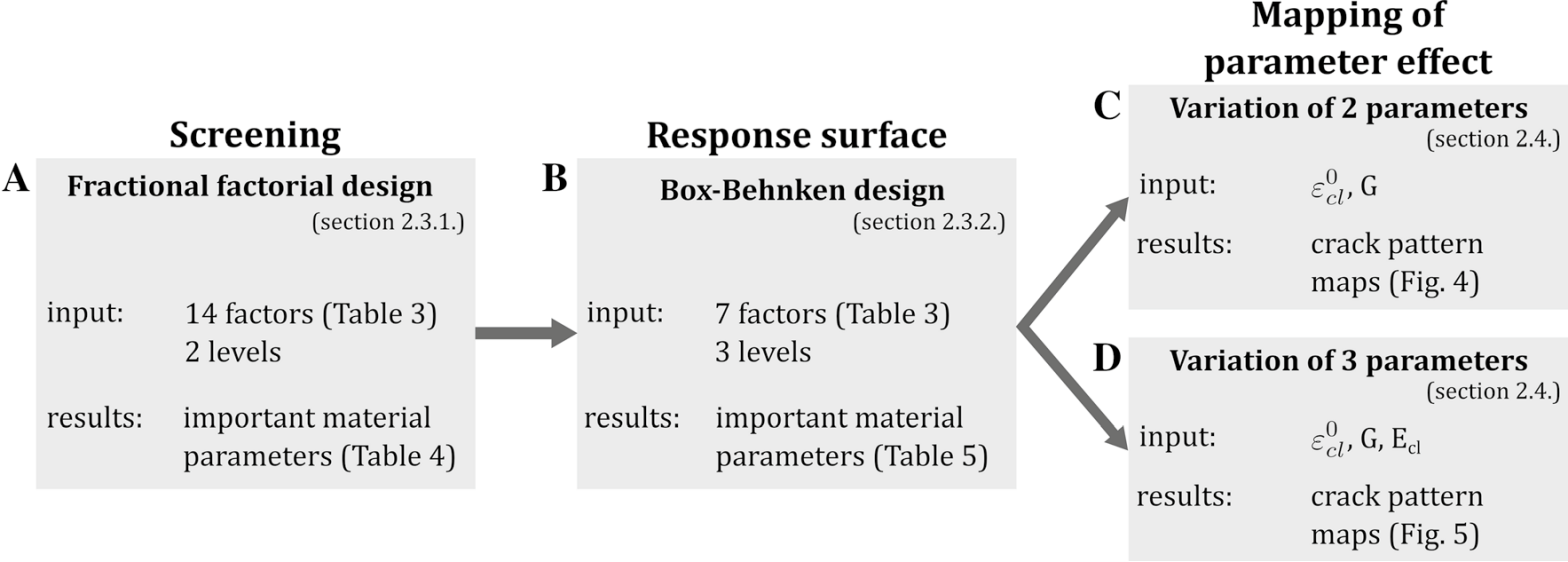
An overview of the analysis procedure

The study design involved a parameter study in three steps, where important parameters were identified in each step and selected for further analysis. An overview of the procedure is given in Fig. 1. The 2D XFEM models introduced in Gustafsson et al. (2018a) were used to simulate crack propagation around an osteon. An initial screening analysis including all 14 material parameters from Gustafsson et al. (2018a) was performed to identify the most influential material parameters through analysis of variance (ANOVA). A subset of 7 parameters were then analyzed using a response surface design, where nonlinear dependencies and interactions between parameters were evaluated. The final step consisted in mapping the effect of material toughness, critical interface strains and cement line stiffness on the crack pattern.

### Cortical bone damage model

**Fig. 2:**
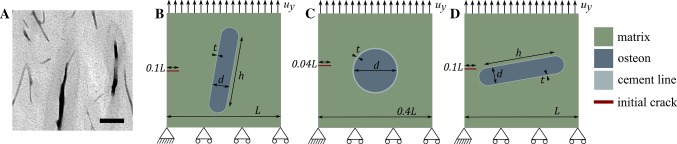
**a** Longitudinal osteons in bovine bone imaged with micro-CT. The average value of a 100-µm-thick image stack is shown in order to visualize the osteonal microstructure. The pixel size is 3 µm, and the scale bar is 200 µm. Model geometries and boundary conditions for the **b** longitudinal, **c** radial and **d** transversal models. In all models, the osteon was embedded in a square matrix and surrounded by the cement line interface separating the osteon from the interstitial matrix. The initial crack length was 10% of the side of each model. In the longitudinal and transversal model, the osteon was modeled as a rectangle rotated 10° with two half circles as endpoints, and in the radial model the osteon was modeled as a circle. The model dimensions were *L *=1 mm, *h* = 650 µm, *d* = 150 µm, *t* = 5 µm, and the thickness of the models was 100 µm

A damage model was recently developed for cortical bone to simulate crack propagation around an osteon at the microscale using XFEM (Gustafsson et al. 2018a). Three models were created representing the cortical bone microstructure with different orientations, where each model comprised one osteon embedded in an interstitial matrix and surrounded by a cement line interface. The Haversian canals were neglected in this study and filled with osteon material, as they were previously shown to induce unrealistic effects in a 2D model (Gustafsson et al. 2018a). All microstructural features, i.e., interstitial matrix, osteons and cement lines, were modeled as linear isotropic elastic materials. The models are referred to as longitudinal, radial and transversal models, according to the orientation of the osteon (Fig. 2). Plane stress 4-node bilinear elements with reduced integration (CPS4R) were used and longitudinal, radial and transversal models contained 27,116, 11,915 and 18,555 elements, respectively. The initial cracks were inserted in the left edge of all models. Tensile tests until failure were simulated using displacement-controlled loading in a quasi-static analysis. Boundary conditions and model dimensions are illustrated in Fig. 2. More detailed information about the models and the mesh design, including a convergence study, can be found in Gustafsson et al. (2018a).

Crack propagation was modeled using the XFEM framework implemented in Abaqus Standard (v2017, Dassault Systemes) with the cohesive segments approach. Damage initiation in matrix and osteon was modeled using the maximum principal strain criterion (MAXPE). The fracture criterion $f$ is defined as

$$ f_{\text{MAXPE}} = \left\{ {\frac{{\varepsilon_{\hbox{max} } }}{{\varepsilon_{\hbox{max} }^{0} }}} \right\} $$

where $\varepsilon_{\hbox{max} }$ is the maximum principal strain and $\varepsilon_{\hbox{max} }^{0}$ is the critical damage initiation strain. Damage is initiated when $f_{\text{MAXPE}} > 1$ and the crack normal is given by the maximum principal strain orientation.

For the cement line, the interface damage model proposed in Gustafsson et al. (2018a) was used. In this model, the MAXPE criterion is used to model crack propagation through the cement line and the quadratic nominal strain criterion (QUADE) is used to model interface damage that causes crack deflection along the interface. Both criteria were implemented in the user-defined damage initiation subroutine UDMGINI, and damage was initiated when $\hbox{max} \left( {f_{\text{QUADE}} , \;f_{\text{MAXPE}} } \right) > 1$. The QUADE fracture criterion was defined as

$$ f_{\text{QUADE}} = \left\{ {\frac{{\varepsilon_{n} }}{{\varepsilon_{n}^{0} }}} \right\}^{2} + \left\{ {\frac{{\varepsilon_{s} }}{{\varepsilon_{s}^{0} }}} \right\}^{2} $$

where $\varepsilon_{n}$ is the normal strain, $\varepsilon_{s}$ is the shear strain and $\varepsilon_{n}^{0}$ and $\varepsilon_{s}^{0}$ denote the critical interface strains in the cement line. A vector oriented perpendicular to the cement line interface was used as crack normal for the QUADE criterion.

For all damage criteria, the damage evolution was assumed to be mode independent and the degradation of the cohesive crack was modeled with an energy-based evolution law. A linear softening behavior was assumed for the traction–separation response inside the cohesive crack, and the strain energy release rate G was determined as the area under the traction–separation curve. The strain energy release rate (material toughness) corresponds to the fracture energy needed to completely open the crack.

### Literature study

A literature study was performed to estimate the range of values for each model parameter (Table 1). Values from experimental and numerical studies were separated to illustrate commonly used model parameters in relation to available experimental data. In cases where quantitative experimental data were not available, relative numbers have been reported. No experimental data exist comparing critical strains in the cement line to critical strains for damage initiation in matrix and osteons; instead, data were reported in terms of interfacial shear strength from osteon push-out tests.

**Table 1:** Compilation of material parameters from experimental and numerical studies in the literature

Material parameter	Microstructure	Abbreviations	Experimental studies	Numerical studies
Young’s modulus (GPa)	Matrix	*E*_mat_	13.8–15.3^a^; 10.8^b^	14.122^v,w,x,y^; 14^z^; 14.6^aa,ab,ac^; 14.6–15.4^ad^; 25.8^ae^; 10^af;^ 4^ag^; 18.5–27.1^ah^
	Osteon	*E*_ost_	(0.63–0.76) * *E*_mat_^c^; 0.91 * *E*_mat_^d^; ~ 0.85 * *E*_mat_^e^; 7.4–18.5^f^	9.13^v,x,z^; 9^z^; 13.5^aa,ab^; 13.5–14.3^ad^; 12.85^w^; 22.5^ae^; 4.4^ag^; 16.6–25.1^ah^; 13.3^ac^
	Cement line	*E*_cl_	*E*_cl_ > *E*_mat_^g,h^; *E*_cl_ < *E*_mat_^i^; 0.7 * *E*_mat_^j^	6.85^v,x^; 7^z^; 10.12^aa,ab^; 0.75 * *E*_ost_^ad^; 9.64^w^; 3.3^ag^; 0.09^ac,ai^
Poisson’s ratio	Matrix	*ν*_mat_		0.153^v,w,x^; 0.3^aa,ab,ae,ag^; 0.15^z,af^; 0.24–0.33^ah^
	Osteon	*ν*_ost_		0.17^v,w,x,z^; 0.33^aa,ab^; 0.3^ae,ag^; 0.24–0.33^ah^
	Cement line	*ν*_cl_		0.49^v,w,x,y,z^; 0.41^aa,ab^; 0.3^ag^
Critical damage initiation strain (MAXPE criterion)	Matrix	$\varepsilon_{{\text{max},{\text{mat}}}}^{0}$	~ 0.01^k^; 0.005^l,m^; 0.011^n^	0.004^v,x,y,aa,ab,ad^; 0.0065^w^; $\left( {\sigma_{\text{mat}}^{0} = 0.55*\sigma_{\text{ost}}^{0} } \right)^{\text{ac}}$
	Osteon	$\varepsilon_{{\text{max},{\text{ost}}}}^{0}$		0.004^v,x,y,aa,ab,ad^; 0.0065^w^;
	Cement line	$\varepsilon_{{\text{max},{\text{cl}}}}^{0}$		0.004^v,x,y,aa,ab,ad^; 0.0065^w^; $\left( {\sigma_{\text{cl}}^{0} = 0.06*\sigma_{\text{ost}}^{0} } \right)^{\text{ac}}$
Critical interface strain (QUADE criterion)	Cement line	$\varepsilon_{{n,{\text{cl}}}}^{0}$		$\left( {\sigma_{{n,{\text{cl}}}}^{0} = \sigma_{{n,{\text{mat}}}}^{0} } \right) ^{\text{ae}}$
	Cement line	$\varepsilon_{{s,{\text{cl}}}}^{0}$	$\left( {\tau_{{s,{\text{cl}}}}^{0} \sim 0.1*\tau_{{s,{\text{mat}}}}^{0} } \right)^o;$ $\left( {\tau_{{s,{\text{cl}}}}^{0} { \lesssim }\,\tau_{{s,{\text{ost}}}}^{0} } \right)^p$	$\left( {\tau_{{s,{\text{cl}}}}^{0} = 0.12*\tau_{{s,{\text{mat}}}}^{0} } \right)^{\text{ae}}$
Strain energy release rate (kJ/m^2^)	Matrix	*G*_mat_	(~ 0.6–0.8)^q^; (~ 0.05^**T**^–0.2^**L**^)^r^; (~ 0.05–0.2^**K**^)^s^; (0.05–0.13^**K**^)^k^; ~ 0.2^t^	0.238^v,w,x,z,ac,af^; 0.09^**K**,aa^; 0.132^ab^; 0.05^**K**,ae^; 1.16^ah^
	Osteon	*G*_ost_	(*G*_ost_ > *G*_mat_)^u^	0.86^v,w,x,z,ac^; 0.11^**K**,aa^; 0.120^ab^; 0.05^**K**,ae^
	Cement line	*G*_cl_		0.146^v,w,x,z^; 0.05^**K**,aa^; 0.084^ab^; 0.05^**K**,ae^ 0.1629^ac,ag^

The mean value is given in case mean ± SD was reported in the cited article. Where no quantitative data were available, relative measures were reported. Values for damage initiation were considered in experimental data reporting stress intensity or fracture energy (strain energy release rate). The stiffness for the matrix was measured under wet conditions. Symbols used in the table: ~ approximate value interpreted from figures; ^**K**^*G* calculated from a reported stress intensity factor *K*, where *G* = *K*^2^/*E*, assuming *E* = 20 GPa (Koester et al. 2008); ^**T**^transverse osteons (crack parallel to osteons); ^**L**^longitudinal osteons (crack perpendicular to osteons)

^a^Faingold et al. (2014); ^b^Nyman et al. (2006); ^c^Rho et al. (1999); ^d^Rho et al. (2002); ^e^Mullins et al. (2009); ^f^Hengsberger et al. (2002); ^g^Skedros et al. (2005); ^h^Milovanovic et al. (2018); ^i^Burr et al. (1988); ^j^Montalbano and Feng (2011); ^k^Chan et al. (2009); ^l^Gargac et al. (2014); ^m^Gustafsson et al. (2018b); ^n^Sun et al. (2010); ^o^Dong et al. (2005); ^p^Bigley et al. (2006); ^q^Norman et al. (1995); ^r^Zimmermann et al. (2009); ^s^Koester et al. (2008); ^t^Nalla et al. (2005); ^u^Mullins et al. (2009); ^v^Abdel-Wahab et al. (2012); ^w^Li et al. (2013); ^x^Vergani et al. (2014); ^y^Budyn and Hoc (2007); ^z^Baptista et al. (2016); ^aa^Idkaidek and Jasiuk (2017); ^ab^Wang et al. (2017); ^ac^Idkaidek and Jasiuk (2017); ^ad^Budyn et al. (2008); ^ae^Demirtas et al. (2016); ^af^Rodriguez-Florez et al. (2017); ^ag^Giner et al. (2017); ^ah^Mischinski and Ural (2011); ^ai^Nobakhti et al. (2014)

### Parameter studies using the design of experiments approach

The first two steps of the parameter study (Fig. 1a, b) are based on a design of experiments approach using fractional factorial designs which enables the parameters that have the largest influence on the outcome variables to be identified. The method lends itself well to computational studies in biomechanics (Dar et al. 2002; Isaksson et al. 2008, 2009). The parameter set introduced in Gustafsson et al. (2018a) was used as a baseline in this study (Table 3). In total, 14 material parameters (factors) were identified and analyzed: Young’s modulus, Poisson’s ratio, the critical damage initiation strain and the strain energy release rate for matrix, osteon and cement line materials, and the critical normal and shear strains in the cement line interface. For the critical interface strains, the average value between crack penetration and deflection as reported in Gustafsson et al. (2018a) for each osteon orientation was used as baseline value. Each factor was assigned a parameter space that was ± 20% of the corresponding baseline value (Table 3).

The importance of each factor for different outcome parameters was determined using analysis of variance (ANOVA). The evaluated outcome parameters were the maximum force (peak value in load curve), fracture energy, crack length (from the coordinates of all crack segments) and crack score. The crack score was used to categorize the crack pattern by assigning a number between 1 and 5 to the crack pattern according to Table 2.

**Table 2:** Crack scores from 1 to 5 used to categorize the different possible crack paths

1	The crack was unaffected by the cement line and propagated straight through the osteon (i.e., $f_{\text{MAXPE}} > f_{\text{QUADE}}$ in the cement line)	
2	The crack was slightly affected by the cement line and deflected a short distance (< 25 μm) into the interface before propagating into the osteon (i.e., $f_{\text{QUADE}} > f_{\text{MAXPE}}$ in a small region)	
3	The crack propagated mostly along the cement line; however, it also propagated through the osteon (i.e., $f_{\text{QUADE}} > f_{\text{MAXPE}}$ in large parts of the cement line)	
4	The crack deflected into the cement line and never penetrated the osteon (i.e., $f_{\text{QUADE}} > f_{\text{MAXPE}}$ in the cement line until the crack entered the matrix on the other side of the osteon)	
5	The crack deflected into the cement line and followed the interface all around the osteon (i.e., $f_{\text{QUADE}} > f_{\text{MAXPE}}$ in all the cement lines)	

The longitudinal model is shown as an example

The importance of the factors was estimated based on the assumption that important factors give a large contribution to the variance. The analysis followed the same rational as presented in Isaksson et al. (2008) where the following parameters needed to be calculated for the analysis of variance. The total sum of squares of the deviation about the mean (SS_*T*_) was calculated as

$$ {\text{SS}}_{T} = \mathop \sum \limits_{i = 1}^{N} \left( {y_{i} - \bar{y}} \right)^{2} $$

where *N* was the number of treatment conditions, *y*_*i*_ the outcome parameter for the *i*th treatment conditions and $\bar{y}$ the mean of all $y_{i}$. The sum of the squares of deviation about the mean for each factor (SS_*F*_) was calculated as

$$ {\text{SS}}_{F} = \mathop \sum \limits_{i = 1}^{n} N_{F,i} \left( {\bar{y}_{F,i} - \bar{y}} \right)^{2} $$

where *n* was the number of levels, $N_{F,i}$ was the number of treatment conditions at each level of each factor and $\bar{y}_{F,i}$ was the mean outcome parameter at each level of each factor. The percentage of the total sum of squares for each factor ($\% {\text{TSS}}$) represented the contribution of each factor to the variance and was considered a measure of the importance of each factor.

$$ \% {\text{TSS}} = \frac{{{\text{SS}}_{F} }}{{{\text{SS}}_{T} }}100\% $$

#### Screening design

An initial two-level screening experiment was performed on longitudinal, radial and transversal models to determine the most important factors (**X**_**screen**_ in Table 3, Fig. 1a). A Resolution IV Two-Level fractional factorial design was used, where the main effects were assumed to be clear of two-factor interactions (Montgomery 2005). The Resolution IV array contained 14 factors (material parameters) and 32 treatment conditions (simulations with different combinations of factors) and was generated with the software JMP (see Supplementary Table 1).

**Table 3:** Material parameters used in the screening experiment with two levels (low and high) and 14 factors (**X**_**screen**_) and the Box–Behnken surface design with three levels (low, baseline and high) and 7 factors (**X**_**BB**_)

Material parameter	Microstructure	Abbreviations	Factor levels			**X**_**screen**_	**X**_**BB**_
			Low (− 20%)	Baseline	High (+ 20%)		
Young’s modulus (GPa)	Matrix	*E*_mat_	12	15	18	1	–
	Osteon	*E*_ost_	9.6	12	14.4	2	1
	Cement line	*E*_cl_	14.4	18	21.6	3	2
Poisson’s ratio	Matrix	*ν*_mat_	0.24	0.3	0.36	4	–
	Osteon	*ν*_ost_	0.24	0.3	0.36	5	–
	Cement line	*ν*_cl_	0.24	0.3	0.36	6	–
Critical damage initiation strain (MAXPE criterion)	Matrix	$\varepsilon_{{\text{max},{\text{mat}}}}^{0}$	0.0032	0.004	0.0048	7	–
	Osteon	$\varepsilon_{{\text{max},{\text{ost}}}}^{0}$	0.0032	0.004	0.0048	8	–
	Cement line	$\varepsilon_{{\text{max},{\text{cl}}}}^{0}$	0.0032	0.004	0.0048	9	3
Critical interface strain (QUADE criterion)	Cement line	$\varepsilon_{{n,{\text{cl}}}}^{0}$	0.00084^L^	0.00105^L^	0.00126^L^	10	4
			0.0012^R^	0.0015^R^	0.0018^R^		
			0.0028^T^	0.0035^T^	0.0042^T^		
	Cement line	$\varepsilon_{{s,{\text{cl}}}}^{0}$	0.00084^L^	0.00105^L^	0.00126^L^	11	4
			0.0012^R^	0.0015^R^	0.0018^R^		
			0.0028^T^	0.0035^T^	0.0042^T^		
Strain energy release rate (kJ/m^2^)	Matrix	*G*_mat_	0.16	0.2	0.24	12	5
	Osteon	*G*_ost_	0.16	0.2	0.24	13	6
	Cement line	*G*_cl_	0.16	0.2	0.24	14	7

Critical interface strains are specified for each osteon direction: ^L^longitudinal, ^R^radial, ^T^transversal. Baseline values are based on values from the literature (Table 1) and introduced in a previous study (Gustafsson et al. 2018a)

#### Response surface design

Next, a response surface design was used to evaluate potential interactions and to further analyze important parameters identified in the screening experiment for the longitudinal and radial models (Fig. 1b). A subset of factors was identified based on their importance for fracture energy, crack length and crack score in the screening study, as these were assumed to be of most importance for the global fracture resistance of bone tissue. A Box–Behnken design was used with 7 factors (**X**_**BB**_ in Table 3), three levels and 62 treatment conditions. The three levels were created by adding the baseline value as a middle value between the low and high levels (Table 3). The Box–Behnken surface design array was generated with the software JMP (see Supplementary Table 2). The critical interface normal and shear strains were in this case assumed to be equal.

### Mapping of parameter effect

The final step was to map the effect of two (Fig. 1c) and three (Fig. 1d) important parameters on the crack pattern while keeping all other parameters at baseline values. The analyzed parameter values spanned a wider range compared to values used in the screening and response surface designs in order to capture the full model behavior as described in Table 2. First, the effect of the material toughness (strain energy release rate) and the critical interface strain was evaluated (Fig. 1c). To reduce the number of varied parameters, both the material toughness parameters and the critical interface parameters were grouped and varied simultaneously (i.e., *G* = *G*_mat_ = *G*_ost_ = *G*_cl_ and $\varepsilon_{\text{cl}}^{0} = \varepsilon_{{n,{\text{cl}}}}^{0} = \varepsilon_{{s,{\text{cl}}}}^{0}$). The material toughness was varied from 0.05 to 0.4 kJ/m^2^ and the critical interface strains from 1e−5 to 0.0045. Simulations were run using all three osteon orientations. Next, the effect of the cement line stiffness was also included (Fig. 1d) and evaluated at two levels: equal to the matrix stiffness (*E*_cl_ = *E*_mat_ = 15 GPa) and 20% lower than the osteon stiffness (*E*_cl_ = 0.8·E_ost_ = 9.6 GPa). Simulations were run using the longitudinal model, and the material toughness was varied from 0.05 to 0.4 kJ/m^2^ and the critical interface strains from 0.0005 to 0.003.

## Results

### Parameter studies using the design of experiments approach

#### Screening design

ANOVA was used to identify the most important factors for four outcome criteria (maximum force, fracture energy, crack length and crack score) in three different model geometries representing longitudinal, radial and transversal osteons (Table 4, contributions higher than 5% are shown in bold font). *E*_mat_ and $\varepsilon_{{\text{max},{\text{mat}}}}^{0}$ were important only for the maximum force outcome and therefore not considered for the response surface design. The fracture energy outcome was dominated by the strain energy release rate *G* of the different materials. Differences were observed when comparing the fracture energy outcome for the different osteon orientations, where *G*_mat_ was most important for the longitudinal models and *G*_cl_ for the transversal models. For the radial model, also *G*_ost_ was found to be important. The crack length and crack score outcomes showed similar results where *E*_cl_, $\varepsilon_{{\text{max},{\text{cl}}}}^{0}$, $\varepsilon_{{s,{\text{cl}}}}^{0}$ and $\varepsilon_{{n,{\text{cl}}}}^{0}$ were important factors. However, $\varepsilon_{{s,{\text{cl}}}}^{0}$ scored high for longitudinal and radial models, whereas $\varepsilon_{{n,{\text{cl}}}}^{0}$ was most important for the transversal model. *E*_ost_ also influenced the crack length and crack score in the radial and transversal models. Poisson’s ratios for the different materials were found to have no or very low importance for all evaluated outcomes. Factors included in Table 4 could explain 57–96% of the variation in the evaluated outcome criteria. In total, seven factors were identified as important for the longitudinal and radial models (*E*_ost_, *E*_cl_, $\varepsilon_{{\text{max},{\text{cl}}}}^{0}$, $\varepsilon_{{s,{\text{cl}}}}^{0}$, *G*_mat_, *G*_ost_ and *G*_cl_) and included in the response surface design (**X**_**BB**_ in Table 3).

**Table 4:** Importance of each factor based on screening experiment evaluated for four different outcome criteria: maximum force, fracture energy, crack length and crack score

ANOVA (%TSS)		Maximum force			Fracture energy			Crack length			Crack score		
**X**_**screen**_	Factors	Long	Radial	Trans	Long	Radial	Trans	Long	Radial	Trans	Long	Radial	Trans
1	*E*_mat_	48.0*	30.2*	8.5*	0.7	0.1	2.8	3.2	0.6	0.1	7.5*	0.1	0.5
2	*E*_ost_	2.6*	8.9*	12.9*	3.6	3.5	0.6	4.7	13.4*	**7.8**	2.7	17.5*	4.2
3	*E*_cl_	0.1	1.3	15.8*	**7.9**	3.7	1.2	**15.6**	22.0*	14.5*	24.4*	17.5*	**8.7**
4	*v*_mat_	0.7	1.4	2.3	0.9	0.1	0.5	0.9	0.3	0.3	2.7	1.5	0.1
5	*v*_ost_	0.0	0.7	0.3	0.0	0.8	0.3	1.1	0.0	0.2	0.3	1.5	0.1
6	*v*_cl_	0.4	0.2	1.6	0.1	0.1	0.3	0.0	0.2	1.0	0.3	3.0	0.1
7	$\varepsilon_{{\rm{max},{\text{mat}}}}^{0}$	39.0*	44.8*	**7.0**	0.9	0.7	3.4	2.2	0.0	0.0	0.3	1.5	0.1
8	$\varepsilon_{{\rm{max},{\text{ost}}}}^{0}$	0.9	2.5*	1.9	2.7	0.0	0.4	2.6	0.7	1.6	2.7	3.0	0.5
9	$\varepsilon_{{\rm{max},{\text{cl}}}}^{0}$	0.0	0.3	**5.1**	**14.9**	3.9	0.1	**25.1**	16.8*	41.9*	24.4*	26.8*	**37.4**
10	$\varepsilon_{{n,{\text{cl}}}}^{0}$	0.0	0.3	**6.4**	0.0	0.0	1.4	0.0	0.0	15.7*	0.3	0.5	**11.5**
11	$\varepsilon_{{s,{\text{cl}}}}^{0}$	0.1	0.5	1.5	4.5	3.5	0.8	**10.9**	12.6*	**5.7**	14.8*	13.7*	4.2
12	*G*_mat_	4.3*	0.0	2.1	37.5*	46.7*	0.7	0.2	0.3	0.2	0.3	0.1	0.1
13	*G*_ost_	0.0	0.2	2.7	0.5	19.6*	9.6*	0.0	2.7	0.3	0.3	4.9	4.2
14	*G*_cl_	0.1	0.5	0.0	4.5	3.1	35.2*	0.8	1.1	0.0	2.7	0.1	0.5
Total sum (%)		96.2	91.8	68.1	78.7	85.8	57.3	67.3	70.7	89.3	83.7	91.7	72.2

The results are given for models with longitudinal, radial and transversal osteons. Significant results in the ANOVA analysis (*p* < 0.05) are indicated with *, and contributions higher than 5% are shown in bold font

#### Response surface design

Results from the response surface design experiment (Table 5) confirmed the trends observed in the screening experiment (Table 4). The interactions scored low in general, and significant interactions involved factors that were already identified as important. The exception was the *G*_cl_ · *G*_cl_ interaction that explained over 7% of the variation for crack length in the radial model, while *G*_cl_ alone was less important. Factors and interactions included in Table 5 could explain 85–92% of the variation in all outcome criteria.

**Table 5:** Importance of each factor from the Box–Behnken surface design evaluated for three different outcomes: fracture energy, crack length and crack score

ANOVA (%TSS)		Fracture energy		Crack length		Crack score	
**X**_**BB**_	Factors	Long	Radial	Long	Radial	Long	Radial
1	*E*_ost_	0.8	0.8	1.3	1.7*	2.5*	2.9*
2	*E*_cl_	7.8*	8.7*	12.6*	22.2*	15.8*	21.1*
3	$\varepsilon_{{\rm{max},{\text{cl}}}}^{0}$	32.5*	6.5*	50.7*	14.8*	44.0*	19.0*
4	$\varepsilon_{\text{cl}}^{0} = \varepsilon_{{n,{\text{cl}}}}^{0} = \varepsilon_{{s,{\text{cl}}}}^{0}$	7.4*	9.4*	11.7*	23.3*	18.0*	36.6*
5	*G*_mat_	31.6*	31.0*	0.3	0.0	0.6	0.0
6	*G*_ost_	0.9	9.0*	0.0	0.0	0.0	0.0
7	*G*_cl_	2.2*	7.3*	0.0	0.0	0.0	0.0
1·3	$E_{\text{ost}} \cdot \varepsilon_{{\rm{max} ,{\text{cl}}}}^{0}$	0.0	2.1*	0.0	4.2	0.8*	0.7
2·3	$E_{\text{cl}} \cdot \varepsilon_{{\rm{max} ,{\text{cl}}}}^{0}$	0.0	2.2*	0.0	**5.2**	1.9*	0.7
2·4	$E_{\text{cl}} \cdot \varepsilon_{\text{cl}}^{0}$	0.1	1.6*	0.1	3.7	0.8*	1.6*
3·4	$\varepsilon_{{\text{max} ,{\text{cl}}}}^{0} \cdot \varepsilon_{\text{cl}}^{0}$	0.1	2.1*	0.1	**5.2**	3.4*	0.7
3·3	$\varepsilon_{{\text{max},{\text{cl}}}}^{0} \cdot \varepsilon_{{\rm{max},{\text{cl}}}}^{0}$	4.5*	0.3	7.4*	0.8	1.0*	0.0
4·4	$\varepsilon_{\text{cl}}^{0} \cdot \varepsilon_{\text{cl}}^{0}$	1.7*	1.2*	2.8*	3.0*	0.3	0.0
7·7	*G*_cl_ · *G*_cl_	0.5	3.2*	0.8	7.1*	2.9*	4.4*
Total sum (%)		90.0	85.3	87.8	91.3	92.1	87.6

Results are shown for model geometries in longitudinal and radial orientations. Significant results in the ANOVA analysis (*p* < 0.05) are indicated with *, and scores higher than 5 are shown in bold font. Interactions with a higher contribution than 3.0% are included in the table

**Fig. 3:**
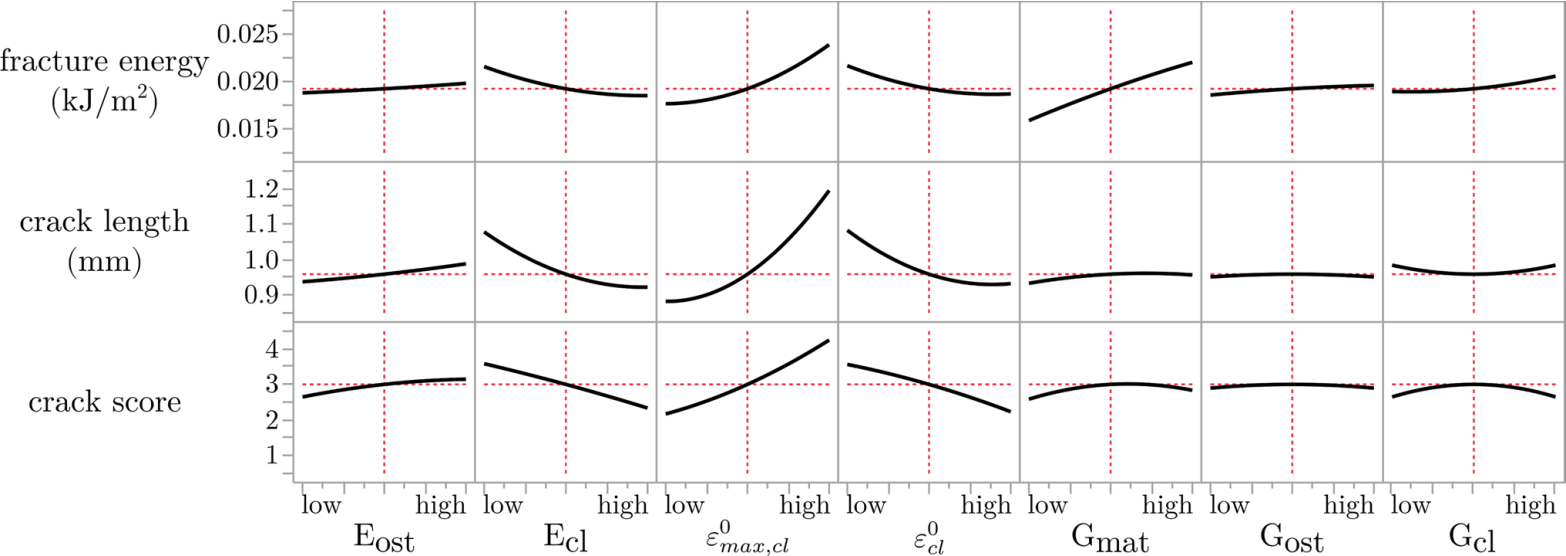
Prediction profiles from the Box–Behnken analysis shown for the longitudinal model. The red lines indicate the average output values at the middle factor levels. Similar prediction profiles were generated for radial models (not shown)

The prediction profiles of the seven factors used in the Box–Behnken surface design (Fig. 3) showed minor nonlinearity in several factors. Most importantly, it showed nonlinear behavior for factors associated with the cement line, where no effect was seen for high levels of the factors and sudden high effects were found when the factors were lower than a threshold value. Most obvious was the effect of the cement line on the crack length, where high damage initiation strain and low critical interface strains promoted crack deflection. The material toughness parameters showed modest effects at the evaluated levels.

### Mapping of parameter effect

**Fig. 4:**
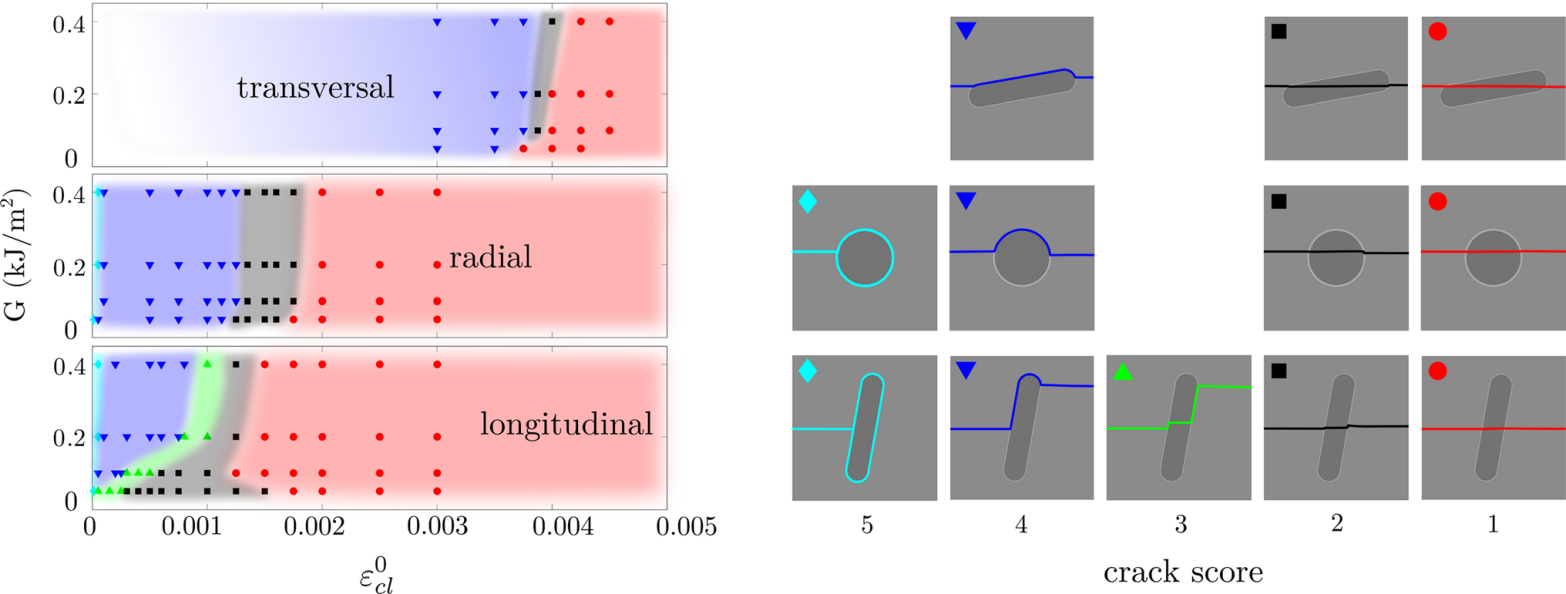
Crack score as a function of the strain energy release rate (*G* = *G*_mat_ = *G*_ost_ = *G*_cl_) and the critical interface strains ($\varepsilon_{\text{cl}}^{0} = \varepsilon_{{n,{\text{cl}}}}^{0} = \varepsilon_{{s,{\text{cl}}}}^{0}$). Each point in the graph corresponds to a simulation, and colored regions are drawn to visualize the region of each crack score. Smaller variations were seen within all crack scores, and the depicted crack trajectories are given as examples for each group

Both the material toughness and the critical interface strains affected the crack trajectory (Fig. 4). In general, low material toughness and high interface strength promoted crack penetration of the osteon, while high toughness and low interface strength promoted crack deflection in the cement line. The different crack patterns (crack scores) were found within continuous regions in Fig. 4, with nonlinear lines marking the borders between the different regions. Lower interface strengths were required for crack deflection in the longitudinal models compared to the transversal. Asymptotic trends reached high toughness values (*G* > 0.2 kJ/m^2^) for all osteon orientations.

**Fig. 5:**
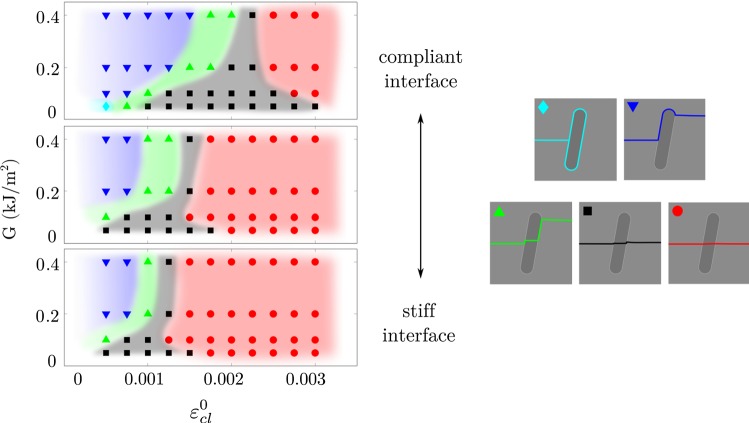
Effect of cement line stiffness in the longitudinal model, where the top plot corresponds to a compliant cement line (*E*_cl_ = 0.8·*E*_ost_ = 9.6 GPa), the middle plot to a cement line with the same stiffness as the matrix (*E*_cl_ = *E*_mat_ = 15 GPa) and the lower plot to a stiff cement line (*E*_cl_ = 1.2·*E*_mat_ = 18 GPa, same as shown in Fig. 3). Each point in the graph corresponds to a simulation, and colored regions are drawn to visualize the region of each crack score, as shown in the right

The effect of the cement line stiffness was seen as a shift in the crack trajectory (Fig. 5), where lower cement line stiffness promoted crack deflection at higher interface strengths. With low cement line stiffness, small deflections were more common (crack score 2, black region in Fig. 5), compared to models with higher cement line stiffness where the crack directly penetrated the interface (crack score 1, red region in Fig. 5).

## Discussion

Local damage properties in cortical bone are difficult to measure experimentally, and hence, material parameters used in computer models are based on very general assumptions and not well established. The aim of this study is to identify important factors for crack propagation in cortical bone and to identify parameters that need further quantification. A comprehensive parameter study was performed using the interface damage model introduced in Gustafsson et al. (2018a), and the importance of 14 material parameters was evaluated in a screening design. This was followed by a response surface design including 7 factors and finally a more in-depth parameter study mapping the effect of a few important parameters. The results emphasized the importance of factors related to the cement line, which are all loosely defined in the literature.

### Parameter studies using the design of experiments approach

**Fig. 6:**
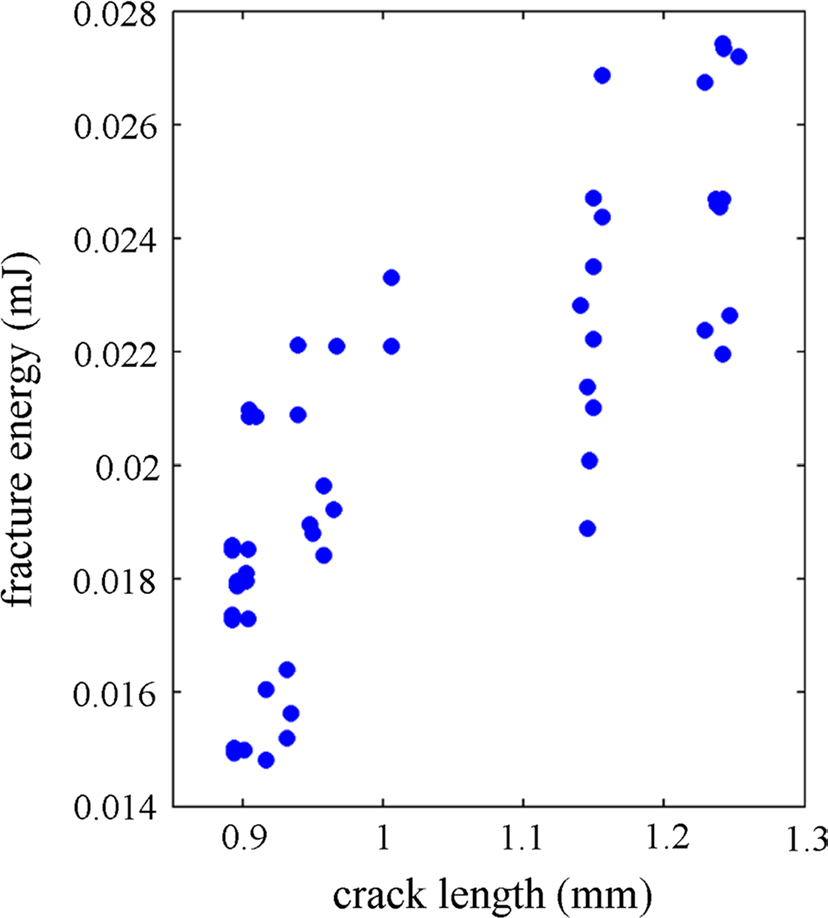
Fracture energy as a function of crack length for all simulations from the Box–Behnken surface design for the longitudinal model

In the initial screening experiment, the maximum force outcome (Table 4) showed that the importance of the osteon and cement line properties was low when the osteon was oriented parallel to the applied load (longitudinal model) and high when oriented perpendicular to the load (transversal model). Similarly, the damage initiation strain in the matrix was a dominant factor in the longitudinal and radial models, while instead all critical strains showed a modest importance (%TSS ~ 5%) in the transverse models. When analyzing the fracture energy and the crack trajectory outcomes, similar trends were seen in the screening study and the Box–Behnken surface design experiment (Tables 4, 5). In the screening study, the material toughness parameters (*G*) were most important for the fracture energy, while in the three-level surface design the cement line parameters also scored high. The Young’s moduli were not important for the fracture toughness (Table 4). This is in line with experiments, showing that the microstructure (osteon density) and tissue porosity are more important for the fracture toughness than the material heterogeneity (stiffness) in cortical bone (Granke et al. 2016). Tissue porosity has been shown to correlate both to crack initiation toughness and to total dissipated energy during fracture at the tissue scale (Granke et al. 2015, 2016). These effects were captured by the critical damage initiation strains and the strain energy release rates for the different materials in the cohesive damage model even though the porosity was not explicitly modeled in this study. At lower length scales, periodic variation in elastic modulus has been shown to increase the fracture toughness (Fratzl et al. 2007). However, as the lamellar bone structure was not included in our models, this effect was also incorporated into the material toughness parameters. Surprisingly, the factors describing the material toughness were not important for the crack length or crack score outcome criteria in the screening and response surface studies. Still, there seemed to be a correlation between crack length and fracture energy (Fig. 6) and this trend was confirmed when analyzing a wide range of material toughness values (Fig. 4). Again, all factors related to the cement line were important for the crack length and the crack score outcomes (Tables 4, 5) and the results were similar when comparing the two outcome criteria. The competition between the crack driving force driving the crack through the osteon and the interfaces trying to deflect the crack, as discussed in Zimmermann et al. (2009, 2010), was illustrated by the prediction profiles (Fig. 3), where high damage initiation strain (high $\varepsilon_{{{\text{max}},{\text{cl}}}}^{0}$) and weak interfaces (low $\varepsilon_{\text{cl}}^{0}$) were both beneficial for crack deflection and increased fracture energy.

As many model parameters lack clear experimental quantification, all factors were varied ± 20% around the baseline value. In this way, all factors were given similar importance, which is beneficial when the range of possible values is not well established. The drawback is that the chosen approach is sensitive to the choice of baseline values and it is possible that another combination of material parameters or another parameter space could display another behavior. As there is no consensus regarding the cement line stiffness (Burr et al. 1988; Milovanovic et al. 2018; Montalbano and Feng 2011; Skedros et al. 2005), the effect of the baseline value for the cement line stiffness was evaluated by repeating the initial screening study for the longitudinal model with *E*_cl_ = *E*_mat_ = 15 GPa. This had no effect on what parameters that were identified as important (data not shown). Furthermore, as both screening and surface design analyses predicted a wide range of different crack paths (crack score 1–4), it indicates that a sufficiently broad and relevant parameter space for capturing the model behavior was chosen.

In our previous study (Gustafsson et al. 2018a), the critical normal and shear strains in the interface were assumed to be equal based on that the weaker of the two critical strains would determine the threshold value for a certain crack trajectory. The effect of this assumption was tested in the screening experiment, which showed that the normal and shear strains were important under different loading conditions (Table 4). The critical normal strain $\varepsilon_{{n,{\text{cl}}}}^{0}$ was important in the transversal model, when the load was applied perpendicular to the cement line interface. The critical shear strain $\varepsilon_{{s,{\text{cl}}}}^{0}$ was important when the load was applied parallel to the interface, as in the longitudinal model. In the remaining simulations performed in this study, the critical normal and shear strains were varied simultaneously (i.e., $\varepsilon_{\text{cl}}^{0} = \varepsilon_{{n,{\text{cl}}}}^{0} = \varepsilon_{{s,{\text{cl}}}}^{0}$), as only one parameter at a time had an effect when separating them for the evaluated model geometries (Table 4).

### Mapping of parameter effect

When evaluating the effect of the critical interface strains and the material toughness on the crack trajectory, we found that the critical interface strains were highly important for the crack score, as crack deflections could always be achieved with a sufficiently low interface strength, independent of the material toughness (Fig. 4). On the other hand, material toughness alone could not be used to enforce crack deflection. This is in agreement with what has been shown using cohesive elements (Mischinski and Ural 2011; Parmigiani and Thouless 2006). However, the material toughness had an effect on the crack trajectory at low levels (*G* ≤ 0.2 kJ/m^2^) and simulations with higher toughness values (up to *G* = 1 kJ/m^2^) were run to confirm that the crack trajectory was not changed (data not shown). The asymptotic behavior for high toughness agreed with the study by Parmigiani and Thouless (2006). Furthermore, the effect of the toughness in each microstructural feature was evaluated by varying the parameters *G*_mat_, *G*_ost_ and *G*_cl_ separately and keeping the others fixed at 0.2 kJ/m^2^ (data not shown). Varying only the matrix toughness gave the same results as varying all toughness parameters simultaneously (as shown in Fig. 4), while no effect was seen on the crack score when varying the material toughness in the osteon or cement line. This illustrates the importance of the matrix toughness and the mechanical state inside the crack when encountering an interface, where cracks in materials with low toughness have a higher risk of penetrating an interface. This aspect was not evaluated by the parameter studies using cohesive elements. Instead, Mischinski and Ural (2011) kept the fracture toughness in the matrix fixed at high values (fracture toughness for normal mode was *G*_nc_ = 1.16 kJ/m^2^ and shear mode *G*_sc_ = 2.97 kJ/m^2^) and Parmigiani and Thouless (2006) modeled a crack already impinging on an interface, not including the effect of the matrix fracture toughness at all. Interestingly, the matrix toughness had large influence in our model at levels corresponding to damage initiation in cortical bone where higher values (*G* ~ 0.2 kJ/m^2^) correspond to young bone and lower values (*G* ~ 0.05 kJ/m^2^) for old bone (Nalla et al. 2004). The reduced material toughness could be one explanation to the straighter cracks seen in old bone (Chan et al. 2009; Koester et al. 2011) as a reduction in toughness would increase the risk of crack penetration of osteons (Fig. 4). This could be highly relevant for understanding crack propagation in cortical bone and changes in fracture resistance due to aging.

The stiffness of the cement line also had an impact on the crack trajectory (Fig. 5), which confirmed the screening results (Tables 4, 5, Fig. 3). With reduced interface stiffness, crack deflections were seen at higher critical interface strains (seen as a shift to the right in Fig. 5). Furthermore, small kinks in the interface (crack score 2) were more common with a more compliant cement line under conditions where direct crack penetrations (crack score 1) occurred in stiffer interfaces (Fig. 5). Nevertheless, experimental findings suggest that interface strength rather than stiffness might be more relevant for determining crack paths in cortical bone. A study at the sub-osteonal scale compared osteonal lamellae and interlamellar areas and reported that both areas had similar mineralization (Katsamenis et al. 2013). Still, cracks preferably propagated through interlamellar areas, even when it meant major deflections from the initial crack path (Katsamenis et al. 2013). Katsamenis et al. (2013) suggested that the orientation of mineralized collagen fibers, rather than the level of mineralization, could be a predictor for the crack path. Similarly, results from osteon push-out tests suggest that the strength of the cement line interface depends on the collagen fiber orientation (Bigley et al. 2006). This means that brittle rather than compliant interfaces could be responsible for deflecting cracks in cortical bone, which is illustrated in Fig. 4. Still, further experimental characterization of the cement line is needed, with focus on the damage properties. Quantification of normal and shear damage properties in the interface would improve the predictive capability of computer models and our understanding of the role of microstructure in cortical bone.

### Model limitations and future studies

Crack propagation, both in experimental setups and in vivo, is a 3D phenomenon that was simplified and analyzed in 2D with three different osteon orientations in this study. The literature considering simulation of crack propagation in bone in full 3D is scarce. Available works focus on analyzing crack initiation as converge problems do not allow the full crack paths to be captured. Models in 3D have been used to simulate crack initiation in femurs (Ali et al. 2014; Marco et al. 2018b), teeth (Zhang et al. 2016) and trabecular bone (Hammond et al. 2019). Another approach was used by Demirtas et al. (2016) who evaluated the possibility to use both XFEM and cohesive elements to simulate crack propagation in cortical bone 3D-geometries. Crack propagation in matrix and osteons was modeled with XFEM but limited to propagate in 2D within a given plane perpendicular to the cortical bone microstructure, while cement lines were modeled with cohesive elements following the cylindrical osteons. An important limitation of that study was that XFEM and cohesive element cracks were not connected; hence, the matrix crack was confined to propagate in the same 2D plane even when cracks followed the cement line out of plane. In all, it is a great challenge to simulate crack propagation in 3D. Our future studies will explore the possibility to extend our modeling framework in 3D to model continuous crack paths in cortical bone at the microscale. The interface damage model can be generalized to 3D by adding the second shear $\varepsilon_{t}$ direction as

$$ f_{\text{QUADE}} = \left\{ {\frac{{\varepsilon_{n} }}{{\varepsilon_{n}^{0} }}} \right\}^{2} + \left\{ {\frac{{\varepsilon_{s} }}{{\varepsilon_{s}^{0} }}} \right\}^{2} + \left\{ {\frac{{\varepsilon_{t} }}{{\varepsilon_{t}^{0} }}} \right\}^{2} $$

but simulating crack propagation in cortical bone, including large crack deflections out of plane, is still beyond the state of the art. In the meantime, important aspects, such as crack deflections and interaction with cortical bone microstructure, can qualitatively be evaluated in 2D.

Another limitation is the simplified material descriptions used in this study, with isotropic linear elastic material models and no open pores. Our previous study showed that Haversian canals exaggeratedly affected the structures in longitudinal and transversal models in 2D, where critical interface strains 50 times lower than the critical matrix strains were required for crack deflection (Gustafsson et al. 2018a). To keep the same model design for all orientations, all Haversian canals were filled with osteon material in this study. The main outcome of the study is not expected to change from excluding the Haversian canals, as relative rather than absolute numbers were of interest when identifying important parameters focusing on the interaction between the crack and the cement line interface. Furthermore, cortical bone displays anisotropic toughening, where the difference in toughness originates from extrinsic toughening mechanisms that are active in different orientations (Koester et al. 2008; Nalla et al. 2004; Zimmermann et al. 2009, 2010). However, less variation is seen when comparing the damage initiation toughness in different directions (Koester et al. 2008), and therefore, the same material toughness values were used in all models. Intrinsic toughening mechanisms (e.g., micro-cracking) appear as plastic mechanisms at higher length scales, and the intrinsic mechanisms occurring in front of the crack tip were included in the damage law describing the cohesive crack. However, new cracks cannot nucleate when there is an active crack in the region. As a result, elements that fulfill the damage criteria away from the propagating crack are not able to fracture, and hence, the stiffness of the structure is overestimated. In complex models with several osteons, this limitation can influence the results, as strain concentrations then can occur at multiple locations in the model. Plastic material formulations for matrix and osteons could then be used to capture more realistic deformation patterns when limited to one single crack and the effect of plasticity could be of interest when looking at the effect of aging. However, for the simple case focusing on crack propagation around one osteon, the use of a simple elastic material formulation is assumed to have a minor impact on the result.

Besides the effect of material parameters on crack propagation around an osteon, there are several geometrical parameters that could be important (e.g., osteon density, size, shape, porosity, cement line thickness etc.). To study the effect of geometrical parameters, more realistic models including multiple osteons are needed. Such complex models are not feasible to use for large screening studies. Instead, the presented parameter study will serve as a useful base when looking in to more realistic scenarios focusing at the effect of aging.

It has been shown repeatedly that cortical bone demonstrates a rising *R*-curve behavior, where the toughness increases with increased crack length (Koester et al. 2008, 2011; Nalla et al. 2004, 2005; Zimmermann et al. 2010). This is due to extrinsic toughening mechanisms, such as crack deflection and crack bridging, which are activated during crack propagation. The toughening effect is largest when the crack propagates perpendicular to the long axis of the osteons (Koester et al. 2008) and diminishes with age (Chan et al. 2009; Koester et al. 2011; Nalla et al. 2004, 2006). An extension of the current models including multiple osteons and realistic osteon geometries will be used to evaluate if the XFEM model can predict a similar rising *R*-curve behavior as seen in experiments with anisotropic effects when comparing different osteon orientations.

## Conclusion

To model crack propagation in cortical bone, it is necessary to use a model that can predict realistic crack paths and capture crack deflections in cement lines. For this, XFEM models are beneficial as they do not need predefined crack paths, and in combination with the proposed interface damage model, realistic crack paths can be simulated. Overall, the cement line properties are crucial for the crack trajectory and both the critical strains in the interface and the stiffness affect the ability to deflect propagating cracks. Further characterization of the interface is needed where also the effect of aging should be investigated. Critical damage strains in the interface have not yet been measured experimentally, and these should be evaluated in relation to critical damage initiation strains in matrix and osteons. The bulk fracture toughness of cortical bone is well documented in the literature (e.g., (Chan et al. 2009; Koester et al. 2008, 2011; Nalla et al. 2004, 2005), and this is assumed to correspond fairly well to the matrix toughness. However, the toughness value for damage initiation should be used for microstructural models instead of the total toughness values that include extrinsic toughening effects. The toughness values for osteons and cement lines have not been well determined experimentally. Our results indicate that these values are important for the total fracture energy dissipated during crack opening but have little importance in determining the crack trajectory. Finally, the results presented in this study illustrate how both the cement line interface and the cohesive state inside the crack when encountering an interface affect the crack trajectory and their interplay could be highly relevant for understanding how aging affects crack propagation and fracture resistance in cortical bone.

## Electronic supplementary material

Below is the link to the electronic supplementary material.

Supplementary material 1 (DOCX 40 kb)
