# Supplementary material for: Crack propagation in cortical bone is affected by the characteristics of the cement line: a parameter study using an XFEM interface damage model
Source: Biomech Model Mechanobiol. 2019 Apr 8;18(4):1247–61. doi: 10.1007/s10237-019-01142-4 (PMC6647448; doi:10.1007/s10237-019-01142-4)
Supplement: Supplementary file 1 — Supplementary material 1 (DOCX 40 kb) [file 10237_2019_1142_MOESM1_ESM.docx]

Supplementary material

**Supplementary Table 1:** The orthogonal array used for the screening experiment, with 14 factors, two levels (-1 and 1) and 32 treatment conditions.

| L_32_ | E_mat_ | E_ost_ | E_cl_ | ν_mat_ | ν_ost_ | ν_cl_ | $\varepsilon_{max, mat}^{0}$ | $\varepsilon_{max, ost}^{0}$ | $\varepsilon_{max, cl}^{0}$ | $\varepsilon_{n,cl}^{0}$ | $\varepsilon_{s,cl}^{0}$ | G_mat_ | G_ost_ | G_cl_ |
| --- | --- | --- | --- | --- | --- | --- | --- | --- | --- | --- | --- | --- | --- | --- |
|  | X1 | X2 | X3 | X4 | X5 | X6 | X7 | X8 | X9 | X10 | X11 | X12 | X13 | X14 |
| 1 | -1 | -1 | -1 | -1 | -1 | -1 | -1 | -1 | -1 | -1 | -1 | -1 | -1 | -1 |
| 2 | -1 | -1 | -1 | -1 | 1 | 1 | 1 | 1 | 1 | 1 | 1 | 1 | -1 | -1 |
| 3 | -1 | -1 | -1 | 1 | -1 | 1 | 1 | 1 | 1 | -1 | -1 | -1 | 1 | 1 |
| 4 | -1 | -1 | -1 | 1 | 1 | -1 | -1 | -1 | -1 | 1 | 1 | 1 | 1 | 1 |
| 5 | -1 | -1 | 1 | -1 | -1 | 1 | 1 | -1 | -1 | 1 | 1 | -1 | 1 | 1 |
| 6 | -1 | -1 | 1 | -1 | 1 | -1 | -1 | 1 | 1 | -1 | -1 | 1 | 1 | 1 |
| 7 | -1 | -1 | 1 | 1 | -1 | -1 | -1 | 1 | 1 | 1 | 1 | -1 | -1 | -1 |
| 8 | -1 | -1 | 1 | 1 | 1 | 1 | 1 | -1 | -1 | -1 | -1 | 1 | -1 | -1 |
| 9 | -1 | 1 | -1 | -1 | -1 | 1 | -1 | 1 | -1 | 1 | -1 | 1 | 1 | -1 |
| 10 | -1 | 1 | -1 | -1 | 1 | -1 | 1 | -1 | 1 | -1 | 1 | -1 | 1 | -1 |
| 11 | -1 | 1 | -1 | 1 | -1 | -1 | 1 | -1 | 1 | 1 | -1 | 1 | -1 | 1 |
| 12 | -1 | 1 | -1 | 1 | 1 | 1 | -1 | 1 | -1 | -1 | 1 | -1 | -1 | 1 |
| 13 | -1 | 1 | 1 | -1 | -1 | -1 | 1 | 1 | -1 | -1 | 1 | 1 | -1 | 1 |
| 14 | -1 | 1 | 1 | -1 | 1 | 1 | -1 | -1 | 1 | 1 | -1 | -1 | -1 | 1 |
| 15 | -1 | 1 | 1 | 1 | -1 | 1 | -1 | -1 | 1 | -1 | 1 | 1 | 1 | -1 |
| 16 | -1 | 1 | 1 | 1 | 1 | -1 | 1 | 1 | -1 | 1 | -1 | -1 | 1 | -1 |
| 17 | 1 | -1 | -1 | -1 | -1 | 1 | -1 | -1 | 1 | -1 | 1 | 1 | -1 | 1 |
| 18 | 1 | -1 | -1 | -1 | 1 | -1 | 1 | 1 | -1 | 1 | -1 | -1 | -1 | 1 |
| 19 | 1 | -1 | -1 | 1 | -1 | -1 | 1 | 1 | -1 | -1 | 1 | 1 | 1 | -1 |
| 20 | 1 | -1 | -1 | 1 | 1 | 1 | -1 | -1 | 1 | 1 | -1 | -1 | 1 | -1 |
| 21 | 1 | -1 | 1 | -1 | -1 | -1 | 1 | -1 | 1 | 1 | -1 | 1 | 1 | -1 |
| 22 | 1 | -1 | 1 | -1 | 1 | 1 | -1 | 1 | -1 | -1 | 1 | -1 | 1 | -1 |
| 23 | 1 | -1 | 1 | 1 | -1 | 1 | -1 | 1 | -1 | 1 | -1 | 1 | -1 | 1 |
| 24 | 1 | -1 | 1 | 1 | 1 | -1 | 1 | -1 | 1 | -1 | 1 | -1 | -1 | 1 |
| 25 | 1 | 1 | -1 | -1 | -1 | -1 | -1 | 1 | 1 | 1 | 1 | -1 | 1 | 1 |
| 26 | 1 | 1 | -1 | -1 | 1 | 1 | 1 | -1 | -1 | -1 | -1 | 1 | 1 | 1 |
| 27 | 1 | 1 | -1 | 1 | -1 | 1 | 1 | -1 | -1 | 1 | 1 | -1 | -1 | -1 |
| 28 | 1 | 1 | -1 | 1 | 1 | -1 | -1 | 1 | 1 | -1 | -1 | 1 | -1 | -1 |
| 29 | 1 | 1 | 1 | -1 | -1 | 1 | 1 | 1 | 1 | -1 | -1 | -1 | -1 | -1 |
| 30 | 1 | 1 | 1 | -1 | 1 | -1 | -1 | -1 | -1 | 1 | 1 | 1 | -1 | -1 |
| 31 | 1 | 1 | 1 | 1 | -1 | -1 | -1 | -1 | -1 | -1 | -1 | -1 | 1 | 1 |
| 32 | 1 | 1 | 1 | 1 | 1 | 1 | 1 | 1 | 1 | 1 | 1 | 1 | 1 | 1 |

**Supplementary Table 2:** Array used for the Box-Behnken surface design experiment, with 7 factors, three levels (-1, 0 and 1) and 62 treatment conditions.

| L_BB_ | E_ost_ | E_cl_ | $\varepsilon_{max, cl}^{0}$ | $\varepsilon_{cl}^{0}$ | G_mat_ | G_ost_ | G_cl_ |
| --- | --- | --- | --- | --- | --- | --- | --- |
|  | X1 | X2 | X3 | X4 | X5 | X6 | X7 |
| 1 | -1 | -1 | 0 | -1 | 0 | 0 | 0 |
| 2 | -1 | -1 | 0 | 1 | 0 | 0 | 0 |
| 3 | -1 | 1 | 0 | -1 | 0 | 0 | 0 |
| 4 | -1 | 1 | 0 | 1 | 0 | 0 | 0 |
| 5 | 1 | -1 | 0 | -1 | 0 | 0 | 0 |
| 6 | 1 | -1 | 0 | 1 | 0 | 0 | 0 |
| 7 | 1 | 1 | 0 | -1 | 0 | 0 | 0 |
| 8 | 1 | 1 | 0 | 1 | 0 | 0 | 0 |
| 9 | 0 | -1 | -1 | 0 | -1 | 0 | 0 |
| 10 | 0 | -1 | -1 | 0 | 1 | 0 | 0 |
| 11 | 0 | -1 | 1 | 0 | -1 | 0 | 0 |
| 12 | 0 | -1 | 1 | 0 | 1 | 0 | 0 |
| 13 | 0 | 1 | -1 | 0 | -1 | 0 | 0 |
| 14 | 0 | 1 | -1 | 0 | 1 | 0 | 0 |
| 15 | 0 | 1 | 1 | 0 | -1 | 0 | 0 |
| 16 | 0 | 1 | 1 | 0 | 1 | 0 | 0 |
| 17 | 0 | 0 | -1 | -1 | 0 | -1 | 0 |
| 18 | 0 | 0 | -1 | -1 | 0 | 1 | 0 |
| 19 | 0 | 0 | -1 | 1 | 0 | -1 | 0 |
| 20 | 0 | 0 | -1 | 1 | 0 | 1 | 0 |
| 21 | 0 | 0 | 1 | -1 | 0 | -1 | 0 |
| 22 | 0 | 0 | 1 | -1 | 0 | 1 | 0 |
| 23 | 0 | 0 | 1 | 1 | 0 | -1 | 0 |
| 24 | 0 | 0 | 1 | 1 | 0 | 1 | 0 |
| 25 | 0 | 0 | 0 | -1 | -1 | 0 | -1 |
| 26 | 0 | 0 | 0 | -1 | -1 | 0 | 1 |
| 27 | 0 | 0 | 0 | -1 | 1 | 0 | -1 |
| 28 | 0 | 0 | 0 | -1 | 1 | 0 | 1 |
| 29 | 0 | 0 | 0 | 1 | -1 | 0 | -1 |
| 30 | 0 | 0 | 0 | 1 | -1 | 0 | 1 |
| 31 | 0 | 0 | 0 | 1 | 1 | 0 | -1 |
| 32 | 0 | 0 | 0 | 1 | 1 | 0 | 1 |
| 33 | -1 | 0 | 0 | 0 | -1 | -1 | 0 |
| 34 | 1 | 0 | 0 | 0 | -1 | -1 | 0 |
| 35 | -1 | 0 | 0 | 0 | -1 | 1 | 0 |
| 36 | 1 | 0 | 0 | 0 | -1 | 1 | 0 |
| 37 | -1 | 0 | 0 | 0 | 1 | -1 | 0 |
| 38 | 1 | 0 | 0 | 0 | 1 | -1 | 0 |
| 39 | -1 | 0 | 0 | 0 | 1 | 1 | 0 |
| 40 | 1 | 0 | 0 | 0 | 1 | 1 | 0 |
| 41 | 0 | -1 | 0 | 0 | 0 | -1 | -1 |
| 42 | 0 | 1 | 0 | 0 | 0 | -1 | -1 |
| 43 | 0 | -1 | 0 | 0 | 0 | -1 | 1 |
| 44 | 0 | 1 | 0 | 0 | 0 | -1 | 1 |
| 45 | 0 | -1 | 0 | 0 | 0 | 1 | -1 |
| 46 | 0 | 1 | 0 | 0 | 0 | 1 | -1 |
| 47 | 0 | -1 | 0 | 0 | 0 | 1 | 1 |
| 48 | 0 | 1 | 0 | 0 | 0 | 1 | 1 |
| 49 | -1 | 0 | -1 | 0 | 0 | 0 | -1 |
| 50 | -1 | 0 | 1 | 0 | 0 | 0 | -1 |
| 51 | 1 | 0 | -1 | 0 | 0 | 0 | -1 |
| 52 | 1 | 0 | 1 | 0 | 0 | 0 | -1 |
| 53 | -1 | 0 | -1 | 0 | 0 | 0 | 1 |
| 54 | -1 | 0 | 1 | 0 | 0 | 0 | 1 |
| 55 | 1 | 0 | -1 | 0 | 0 | 0 | 1 |
| 56 | 1 | 0 | 1 | 0 | 0 | 0 | 1 |
| 57 | 0 | 0 | 0 | 0 | 0 | 0 | 0 |
| 58 | 0 | 0 | 0 | 0 | 0 | 0 | 0 |
| 59 | 0 | 0 | 0 | 0 | 0 | 0 | 0 |
| 60 | 0 | 0 | 0 | 0 | 0 | 0 | 0 |
| 61 | 0 | 0 | 0 | 0 | 0 | 0 | 0 |
| 62 | 0 | 0 | 0 | 0 | 0 | 0 | 0 |
